# The Use of Intravenous Neostigmine in Palliation of Severe Ileus

**DOI:** 10.1155/2013/796739

**Published:** 2013-02-14

**Authors:** Pashtoon Murtaza Kasi

**Affiliations:** International Scholars Program, Department of Internal Medicine, University of Pittsburgh Medical Center (UPMC), Pittsburgh, PA 15213, USA

## Abstract

Neostigmine is a parasympathomimetic drug that acts as a reversible acetylcholinesterase inhibitor. Clinically it is used in patients with acute colonic pseudo-obstruction (ACPO or Ogilvie's syndrome, which is a gastrointestinal motility disorder characterized by marked dilatation of the colon in the absence of mechanical obstruction), postoperative ileus, urinary retention, myasthenia gravis, and in anesthesia to reverse the effects of nondepolarizing muscle relaxants. Both bolus and infusion are noted to be effective and lead to prompt evacuation of flatus or stool with a reduction in abdominal distention on physical examination. Median duration is noted to be 4–30 minutes in some trials. Here we present our experience of using 2 mg of intravenous neostigmine to help relieve the severe abdominal distention and ileus in a patient with severe fecal impaction when all conservative measures had been futile. The most frequent side effect of the drug is abdominal pain/cramping, which was noted in our patient as well. Other complications include bradycardia which is very infrequently symptomatic to require atropine. Overall, the drug is a simple, safe, and effective strategy; and as pointed out in the previous studies, the drug appears to be underused in patients who do not have a true contraindication to its use.

## 1. Case Presentation

A 76-year-old man with a past medical history significant for cerebrovascular accident (CVA) with severe residual right-sided hemiplegia/aphasia (bedbound), vascular dementia, HTN, and depression was admitted to our service from a nursing home because of worsening abdominal distention and pain from severe constipation since several weeks apparently.

Per nursing home records, patient had been having problems with constipation for the last 2-3 weeks. At the nursing facility, they had tried all kinds of oral stool softeners/laxatives including magnesium citrate, docusate, senna, polyethylene glycol, and prune juices followed by enemas (soap suds enemas and fleet enemas) with no results. Prokinetic drugs (including erythromycin and metoclopramide) were also tried. Methylnaltrexone (Relistor) injections were also administered at the facility as well with no outcome. 

Given progressive worsening in his abdominal distention/pain, along with ongoing constipation, patient was brought to our tertiary care facility for further care and management.

**Figure 1:**
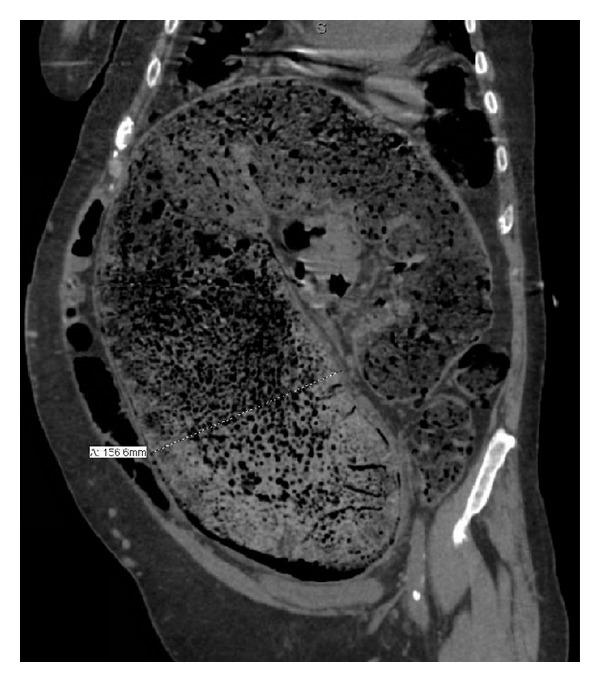
Coronal section on a computerized tomography (CT) scan showing severe fecal impaction. In this particular section, the rectum is noted to be distended up to approximately 16 centimeters.

At the time of admission, a computerized tomography (CT) scan of his abdomen was done which unfortunately revealed very severe fecal impaction (Figure 1). According to radiology, significant fecal impaction was noted essentially in patient's entire colon with the rectum measuring up to 19 cm in transverse diameter. Note was also made of mild wall thickening and perirectal stranding, and that a stercoral ulcer could not be excluded.

Here we present our experience of using intravenous neostigmine to help relieve the severe abdominal distention and ileus when all conservative measures had been futile. 

## 2. Discussion

Neostigmine is a parasympathomimetic drug that acts as a reversible acetylcholinesterase inhibitor. It was first synthesized by Aeschlimann and Reinert in 1931 [1]. By interfering with the breakdown of acetylcholine, neostigmine indirectly stimulates both nicotinic and muscarinic receptors.

Clinically it is used in patients with acute colonic pseudo-obstruction (ACPO or Ogilvie's syndrome, which is a gastrointestinal motility disorder characterized by marked dilatation of the colon in the absence of mechanical obstruction), postoperative ileus, urinary retention, myasthenia gravis, and in anesthesia to reverse the effects of nondepolarizing muscle muscle relaxants [2–5]. Colonic pseudo-obstruction in particular is postulated to occur as an outcome of autonomic imbalance and is usually seen in elderly patients with numerous comorbidities, with an associated high morbidity and mortality [6, 7].

The use of methylnaltrexone has also been reported in patients with acute colonic pseudo-obstruction (ACPO) or Ogilvie syndrome; however, the effectiveness seems to be limited to more so in patients who are on high doses of narcotics [8]. 

“Controlled clinical trials have shown that the acetylcholinesterase inhibitor neostigmine is an effective treatment with initial response rates of 60–90 per cent; other drugs for use in this area are in evolution” [9]. Both bolus and slow infusion are noted to be effective [10].

The most frequent side effect of the drug is abdominal pain/cramping, which was significant in our patient as well. Other complications with the use of neostigmine include bradycardia. In a randomized control trial published in the New England Journal of Medicine in 1999, two patients had symptomatic bradycardia, requiring atropine [11]. One patient in their trial had syncope after walking to the bathroom 30 minutes after administration of the drug, which was a violation of the protocol. Patients are advised to be in bed for several hours after receiving the drug. Otherwise, no other significant complications were noted. 

Amongst the various limited series, the drug seems to be effective, well tolerated, and safe alongside the advantage of providing immediate relief [12–15]. Very rare case reports of cardiac arrest, bowel ischemia/perforations after administration of the drug, however, have been reported [16, 17]. And as pointed out by Loftus and colleagues, the drug appears to be underused in patients who do not have a true contraindication to its use [18].

In our particular patient, over the course of his admission initially, no progress was made. Both gastroenterology and gastrointestinal surgical services were on board. Given the degree of patient's fecal impaction and worsening nature, surgical options were even being considered and endoscopic evacuation was considered not an option. Patient, however, at the same time was not considered a surgical candidate. 

Enemas would be helpful in such a situation to help soften the feces; however, given the apparent chronicity of the problem, administration of enemas was not entirely successful. Pain was also a limiting factor given the presence of stercoral ulcers. At that point in time, we used copious amounts of topical lidocaine jelly and did an extensive manual disimpaction. Following this, administration of enemas became easier and patient was put on a comprehensive bowel regimen. With respect to enema, “soap suds” enemas were alternated with “gastrografin” enemas for the first couple of days; and then soap suds enemas alone given the risk of perforations seen with repeated use of gastrografin enemas [19].

**Figure 2:**
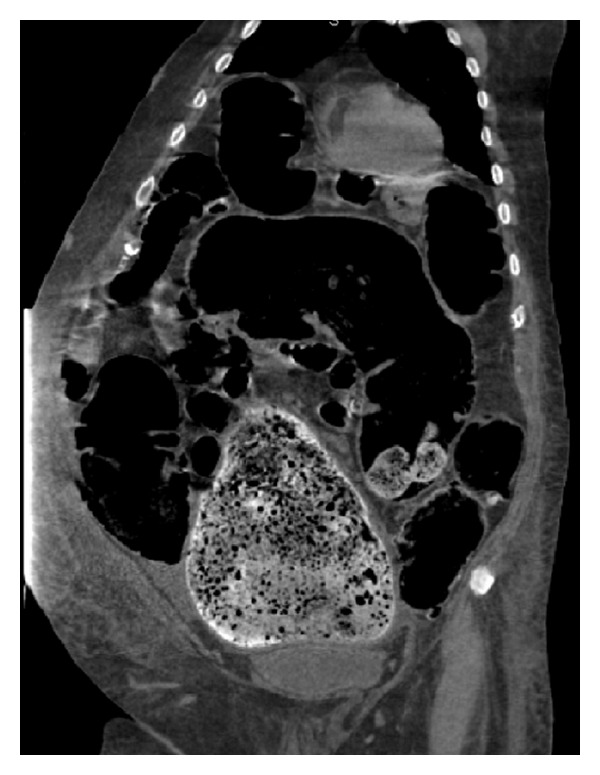
Marked improvement in the fecal impaction but still with persistence of abdominal distention and ileus.

**Figure 3:**
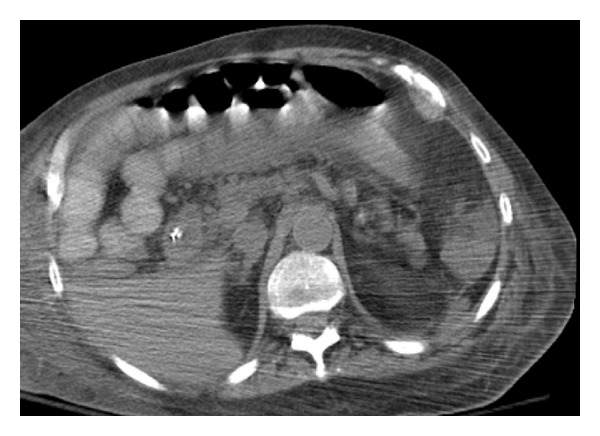
Transverse section on a computerized tomography (CT) scan showing significant relief of the severe impaction and ileus.

Follow-up imaging showed significant improvement in the fecal impaction (Figure 2); however, the abdominal distention and ileus persisted. At that point in time after discussion with the consulting services and pharmacists, neostigmine 2 mg IV was administered slowly over 3-4 minutes. Within 30–45 seconds after administration, patient had immediate relief with prompt evacuation of flatus and stool with a massive reduction in his abdominal distention on physical examination (>5 liters of stool output; liquid/paste with hard stools as well was noted within the first 45 minutes). Following this, patient was incontinent of several bowel movements within the next couple of hours. Clinically, the patient's abdomen, which was hard and distended, within an hour became soft and patient's pain/distress was almost completely relieved (Figure 3). 

No complications were noted. Patient's heart rate was monitored during and after the administration. The heart rate was in the 90 s–100 s at the time of administration and for 5 seconds came down to 60 s; and as soon as patient started having abdominal cramping followed by bowel movements, went back up to the 90 s. Atropine was ready at patient's bedside in case if it was needed. Aspiration precautions were also taken. 

Based on the significant results and relief of distress noted in our particular patient without any complications, we strongly propose the use of neostigmine in similar and other patients with severe abdominal distention/ileus, where all other conservative measures have been futile. However, an important lesson/factor is the initial need for digital rectal examination and manual disimpaction with extensive use of enemas and hydration to help soften the stool; otherwise, jumping to the use of neostigmine directly to induce peristalsis when the rectal impaction has not been relieved can potentially lead to more distress for the patient and possible perforation. Electrolytes should be frequently monitored given the massive losses that occur when the drug is effective. 

## 3. Conclusions

- Neostigmine is a simple, safe, and effective therapy in patients with severe abdominal distention from fecal impaction/ileus.
- An important lesson/factor in the care of patients with severe impaction is the classic teaching of the initial need for digital rectal examination and manual disimpaction with extensive use of enemas and hydration to help soften the stool; otherwise, jumping to the use of neostigmine directly to induce peristalsis when the rectal impaction has not been relieved can potentially lead to more distress for the patient and possible perforation.
- Electrolytes should be frequently monitored given the massive losses that occur when the drug is effective.
